# The Role of Hyperthermia in Methamphetamine-Induced Depression-Like Behaviors: Protective Effects of Coral Calcium Hydride

**DOI:** 10.3389/fnmol.2021.808807

**Published:** 2022-01-04

**Authors:** Xintao Wang, Bonan Tong, Rongji Hui, Congcong Hou, Zilu Zhang, Ludi Zhang, Bing Xie, Zhiyu Ni, Bin Cong, Chunling Ma, Di Wen

**Affiliations:** ^1^College of Forensic Medicine, Hebei Medical University, Shijiazhuang, China; ^2^Hebei Key Laboratory of Forensic Medicine, Collaborative Innovation Center of Forensic Medical Molecular Identification, Shijiazhuang, China; ^3^Research Unit of Digestive Tract Microecosystem Pharmacology and Toxicology, Chinese Academy of Medical Sciences, Shijiazhuang, China; ^4^The First Clinical Medical College of Peking University Health Science Center, Peking University, Beijing, China; ^5^School of Basic Medical Sciences, Hebei University, Baoding, China

**Keywords:** methamphetamine, hyperthermia, depression, coral calcium hydride, oxidative stress, neuroinflammation

## Abstract

Methamphetamine (METH) abuse causes irreversible damage to the central nervous system and leads to psychiatric symptoms including depression. Notably, METH-induced hyperthermia is a crucial factor in the development of these symptoms, as it aggravates METH-induced neurotoxicity. However, the role of hyperthermia in METH-induced depression-like behaviors needs to be clarified. In the present study, we treated mice with different doses of METH under normal (NAT) or high ambient temperatures (HAT). We found that HAT promoted hyperthermia after METH treatment and played a key role in METH-induced depression-like behaviors in mice. Intriguingly, chronic METH exposure (10 mg/kg, 7 or 14 days) or administration of an escalating-dose (2 ∼ 15 mg/kg, 3 days) of METH under NAT failed to induce depression-like behaviors. However, HAT aggravated METH-induced damage of hippocampal synaptic plasticity, reaction to oxidative stress, and neuroinflammation. Molecular hydrogen acts as an antioxidant and anti-inflammatory agent and has been shown to have preventive and therapeutic applicability in a wide range of diseases. Coral calcium hydride (CCH) is a newly identified hydrogen-rich powder which produces hydrogen gas gradually when exposed to water. Herein, we found that CCH pretreatment significantly attenuated METH-induced hyperthermia, and administration of CCH after METH exposure also inhibited METH-induced depression-like behaviors and reduced the hippocampal synaptic plasticity damage. Moreover, CCH effectively reduced the activity of lactate dehydrogenase and decreased malondialdehyde, TNF-α and IL-6 generation in hippocampus. These results suggest that CCH is an efficient hydrogen-rich agent, which has a potential therapeutic applicability in the treatment of METH abusers.

## Highlights

- Hyperthermia plays a key role in METH-induced depression-like behaviors.
- High ambient temperature aggravates METH-induced depressive behaviors.
- CCH pretreatment inhibits METH-induced depression-like behaviors.
- CCH reduces METH-induced hippocampal synaptic plasticity damage.
- CCH has a potential therapeutic applicability in the treatment of METH abusers.

## Introduction

Methamphetamine (METH) is a widely abused psychoactive substance all over the world (De-Carolis et al., 2015). Long-term or large dose use of METH leads to serious abnormalities in the cardiovascular, digestive, and immune system, and especially causes irreversible damage to the central nervous system (CNS) (Papageorgiou et al., 2019). As such, METH abusers are susceptible to neurodegenerative diseases, such as Parkinson’s (Arab et al., 2019), Alzheimer’s (Panmak et al., 2021), and Huntington’s disease (Johnson et al., 2006), and generally present with a variety of psychiatric symptoms, such as depression and schizophrenia (Pogorelov et al., 2012).

Studies have shown that dopamine oxidative stress, excitotoxicity, and neuroinflammation are the most important mechanisms in METH-induced neurotoxicity (Halpin et al., 2014; Shaerzadeh et al., 2018; Pan et al., 2020). Additionally, hyperthermia is also a critical factor, and METH caused hyperthermia occurs in a dose- and ambient temperature-dependent manner (Molkov et al., 2014). A single medium or high dose of METH can cause a rapid rise of core body temperature, which is maintained for several hours, and this persistent hyperthermia aggravates METH-induced oxidative stress, excitotoxicity and neuroinflammation (Matsumoto et al., 2014; Liao et al., 2021). Immunohistochemistry index of tyrosine hydroxylase (Hotchkiss and Gibb, 1980; Kaewsuk et al., 2009), dopamine transporter (Sambo et al., 2018), glial fibrillary acidic protein (Castelli et al., 2014), and c-Fos in mice brain samples taken 6 days after METH administration confirmed that exposure to hot ambient temperature increases the neurotoxicity of METH (Cornish et al., 2012). However, whether hyperthermia plays a role in METH-induced psychiatric symptoms remains elusive.

Increasing evidence has indicated that treatment with antioxidant and anti-inflammatory agents is an effective intervention strategy that can effectively reduce the incidence of METH-induced neurotoxic complications (Murakami et al., 2018; Xie et al., 2018). Molecular hydrogen, as a novel healthcare product for a wide range of diseases, has recently become increasingly popular because of its unique anti-oxidative capability of selectively scavenging highly cytotoxic oxygen radicals and its anti-inflammatory properties (Qi et al., 2021). Our previous studies have revealed that molecular hydrogen delivered by *ad libitum* hydrogen-rich water (HRW) consumption significantly inhibited METH-induced spatial memory impairment in the Barnes and Morris water maze tests (Wen et al., 2019). In addition, hydrogen-rich saline (HRS) injections attenuated symptoms of low dose METH-induced behavioral sensitization (Wen et al., 2020). Up to now, drinking or bathing with HRW and inhalation of hydrogen gas (HG) have been used as the routes for administering hydrogen to humans (Zhu et al., 2018; Mikami et al., 2019). However, these methods hardly lead to the long-term effective accumulation of hydrogen owing to its low solubility in water. Coral calcium hydride (CCH), a porous powder made of coral calcium reacting with hydrogen at high temperature, generates HG gradually, when exposed to water (Ueda et al., 2010). Previous studies have reported that the maximum concentration of HG generated reached nearly 600 ppb in 5 ∼ 10 g CCH/L suspension. Moreover, this hydrogen could be steadily released for at least 24-h before its generation gradually declined (Hou et al., 2016). Herein, we hypothesized that the administration of CCH used to produce pure hydrogen may alleviate hyperthermia and prevent depression-like behaviors caused by high dose of METH exposure.

In this study, animals were treated with METH in different ambient temperatures to investigate the role of hyperthermia in METH-induced depression-like behaviors *via* tail suspension test (TST), forced swimming test (FST), and locomotion test (LMT). In addition, the effect of CCH on METH-induced hyperthermia and depression-like behaviors was also explored. As the damage of hippocampal neurons plays a key role in plasticity regulation of synapses and a critical role in the mechanism of depression, Golgi staining in hippocampus was conducted, and the index of oxidative stress and neuroinflammation were also measured by the detection of lactate dehydrogenase (LDH), malondialdehyde (MDA), IL-6 and TNF-α levels.

## Materials and Methods

### Animals

Three hundred and ninety C57BL/6 mice, initially weighing 20–22 g (8 weeks old) were ordered from Beijing Vital River Laboratory Animal Technology Co., Ltd., China. All the mice were provided with food and water *ad libitum* and were kept in a climate controlled environment, at a consistent temperature (22 ± 1°C), humidity (approximately 60%), and a 12-h light/dark cycle (lights off at 7:00 a.m.). All experimental procedures were approved by the Local Animal Use Committee of Hebei Medical University and performed in accordance with the National Institutes of Health Guide for the Care and Use of Laboratory Animals.

### Drugs

DL-METH was provided by Beijing Municipal Public Security Bureau, China. CCH was provided by Shanghai Quanren Biological Technology Co., Ltd. (Shanghai, China). The stock solution of METH (1 g/mL) was dissolved in 0.9% sterile saline, and CCH was suspended in pure water before use. The concentration of METH was adjusted to an appropriate injection volume of 10 mL/kg of body weight in each experiment.

### Behavioral Testing

#### Tail Suspension Test

The procedure of behavioral testing was consistent with previous studies (Hao et al., 2019; Zhao et al., 2019; Luo et al., 2021). Four brightly lit 20 cm × 20 cm × 35 cm white, plexiglass arenas were used for the TST. The tail of each mouse was attached to a hook placed 3 cm from the top of each box using adhesive tape placed 1 cm away from the tip of the tail for a duration of 6 min. Immobility was defined as the absence of movement of limb or body when hung passively, and the immobility time during the last 5 min were measured. The behavioral tests were videotaped and analyzed using Noldus Video Tracking Software (Wageningen, Netherlands). Animals were separated from each other to prevent visual and acoustic interplay. The arena was cleaned with 75% alcohol between each test.

#### Forced Swim Test

Four transparent resin cylinders with a diameter of 10 cm and a height of 23 cm were filled with 15 cm of 23–25°C warm water for performing the FST. Each mouse was placed in a cylinder and videotaped for 6 min to record the immobility time. The immobility in the last 5 min was measured and analyzed using Noldus Video Tracking Software. Immobility was defined as the absence of limb or body movements, except for what is necessary to keep the body from sinking. During the test, mice were separated from each other to prevent visual and acoustic interplay. The used water was replaced with fresh water after each test.

#### Locomotion Test

The mice were placed in a brightly lit 40 cm × 40 cm white plexiglass arenas. The movement and location of the mice were recorded. The total distance traveled within the arena was recorded for a single 5 min session, which was used to measure the motor ability of the mice. The arena was cleaned with 75% alcohol between each test.

### Experimental Design

#### Effect of Ambient Temperature on Methamphetamine-Induced Hyperthermia and Depression-Like Behaviors

**FIGURE 1:**
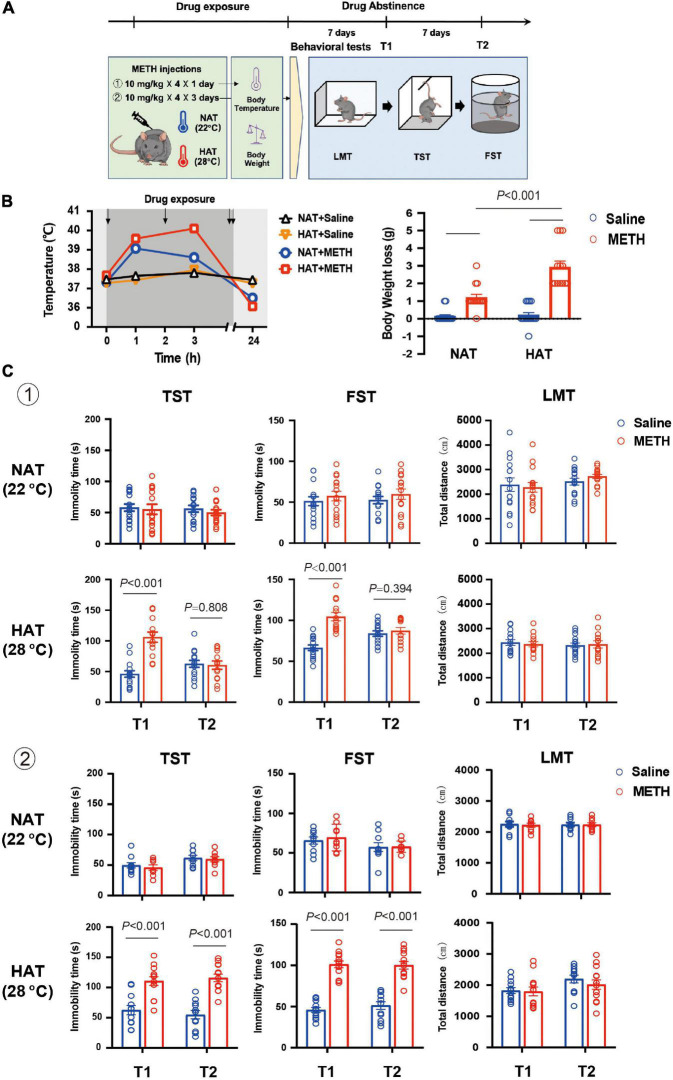
Methamphetamine (METH) exposure induced hyperthermia and depression-like behaviors in an ambient temperature and dose-dependent manner. **(A)** Timeline of drug treatment and behavioral tests. **(B)** High ambient temperature aggravated METH-induced hyperthermia (left) and body weight loss (right). Four doses of METH (10 mg/kg i.p.) treatments were given to mice each within 2-h interval. The arrows represented drug exposure. Body temperature was determined at 0-, 1-, 3-, and 24-h after the first METH treatment, and body weight loss was calculated at 24-h after drug treatment. (*n* = 15, 15, 15, and 13) **(C)** METH exposure (a: 10 mg/kg × 4 injections × 1 day; b: 10 mg/kg × 4 injections × 3 days) under high ambient temperature (28°C) induced depression-like behaviors in mice. The behavioral tests including locomotion test (LMT), tail suspension test (TST), and forced swimming test (FST) were performed 7 (T1) and 14 (T2) days after METH treatments (**a:** *n* = 15 and 15 for NAT, *n* = 15 and 13 for HAT; **b:** *n* = 10 and 9 for NAT, *n* = 12 and 13 for HAT). Data are expressed as the mean ± SEM.

The drug exposure regimen and dose described in previous studies were used in the present study. As shown in Figure 1A, two batches of mice kept at normal ambient temperature (NAT) of 22°C and high ambient temperature (HAT) of 28°C, respectively, were treated with four doses of 10 mg/kg METH *via* intraperitoneal (i.p.) injections with a 2-h interval in between each injection for 1 or 3 days. Two hours after the last METH injection, animals were placed back to their home cages at normal ambient temperature (22°C). The core body temperature of each mouse was recorded at 1-h after the first and second METH injection. Also, the core body temperature and body weight were measured at 24-h after the first METH injection. The depression-like behaviors were tested 7 and 14 days after METH exposure. All mice were tested in the following order: LMT, TST, and FST.

#### Effect of Drug Exposure Regimen and Dose on Methamphetamine-Induced Depression-Like Behaviors

**FIGURE 2:**
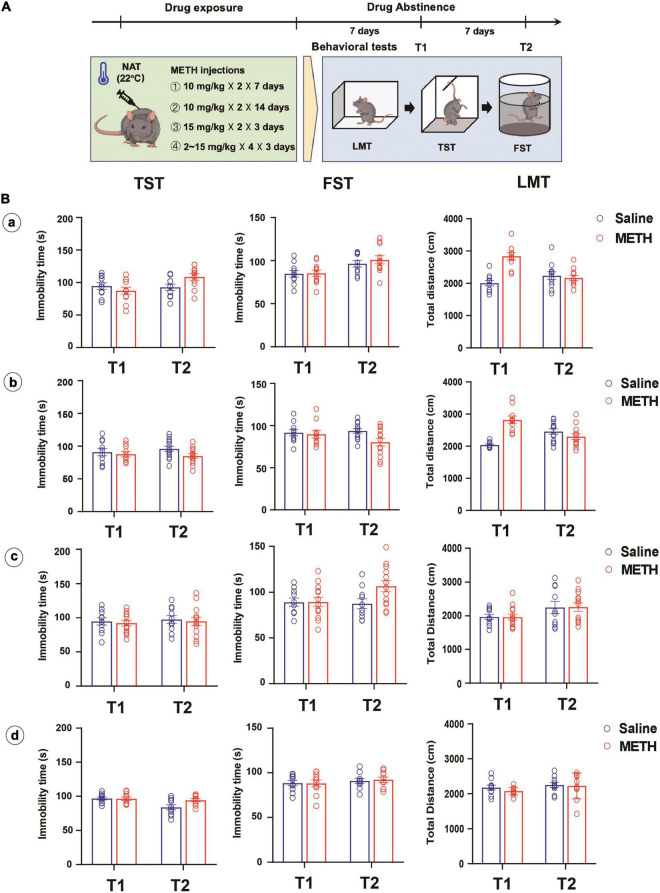
Longer-term (10 mg/kg × 2, 7 and 14 days), higher dose (15 mg/kg × 4, 3 days) or escalating-dose (2–15 mg/kg, 3 days) regimen of METH treatments under normal ambient temperature (22°C) failed to induce depression-like behaviors in mice. **(A)** Timeline of drug treatment and behavioral tests. **(B)** Different METH treatment procedures (**a:** 10 mg/kg × 2, 7 days; **b:** 10 mg/kg × 2, 14 days; **c:** 15 mg/kg × 4, 3 days; **d:** 2, 2, 5, 5, 5, 5, 10, 10, 10, 10, 15, and 15 mg/kg in 3 days) were performed in mice, and the depression-like behaviors and locomotion were tested at 7 (T1) and 14 (T2) days after METH exposure (**a:** *n* = 10 and 11; **b:** *n* = 10 and 12; **c:** *n* = 10 and 13; **d:** *n* = 10 and 10). Data are expressed as the mean ± SEM.

Subsequently, the effect of different drug exposure regimens and doses on METH-induced depression-like behaviors was investigated (Figure 2A). Two batches of mice were administered 14 and 28 doses of 10 mg/kg METH injections (twice per day with 2-h interval) over 7 and 14 days, respectively. One batch was given four 15 mg/kg METH injections with 2-h interval for 3 days and one batch was given gradually increasing doses (2, 2, 5, 5, 5, 5, 10, 10, 10, 10, 15, and 15 mg/kg) of METH in 3 days (Martins et al., 2011; Ding et al., 2020; Chen et al., 2021). These experiments were all performed under NAT of 22°C. The depression-like behaviors were tested 7 and 14 days after METH exposure.

#### Effect of Molecular Hydrogen Generated by Coral Calcium Hydride on Methamphetamine-Induced Hyperthermia and Depression-Like Behaviors

Mice were pre-treated with CCH (100 and 200 mg/kg, intragastric route [i.g.]) resuspended in 0.2 mL pure water 1-h before METH exposure (10 mg/kg, once) at 28°C ambient temperature. The core body temperature was determined every 20 min for 2-h. To explore the effect of CCH on METH-induced depression-like behaviors, mice were treated with METH (10 mg/kg × 4) for 3 days and then administered with pure water (0.2 mL, i.g.) or CCH (100 and 200 mg/kg, i.g.) twice per day. The depression-like behaviors were tested 7 and 14 days after METH exposure by TST, FST, and LMT.

#### Effect of Methamphetamine Exposure and Coral Calcium Hydride Administration on the Hippocampal Synaptic Plasticity and Levels of Oxidative Stress Products and Inflammatory Cytokines

Mice were treated with METH (10 mg/kg × 4) for 3 days and then administered with pure water (0.2 mL, i.g.) or CCH (100 and 200 mg/kg, i.g.) twice per day. Golgi staining was performed to examine the effect of METH exposure and CCH administration on the hippocampal synaptic plasticity. Hippocampal tissues were dissected to detect the level of oxidative stress products (MDA and LDH) and inflammatory cytokines (TNF-α and IL-6) by commercial assay kits.

### Golgi Staining

Golgi staining was used to detect the changes of dendritic spines in hippocampal neurons. The commercial Golgi staining kit made by Genmed Medicine Technology Co., Ltd. (Shanghai, China) was used. The mice were anesthetized with isoflurane and perfused with 1 × PBS solution and 4% paraformaldehyde. The brains of these mice were harvested and washed with pure water, then placed in a soak solution for 2 weeks in the dark and subsequently transferred to 30% sucrose solution for 48-h. Sagittal sections (80 ∼ 100 μm thick) were stained using the oscillating sectioning technique. One out of every five sections were selected, and a total of three sections were selected from each mouse. After Golgi staining, the changes of dendritic spines were observed under a microscope, and the number of dendritic spines was calculated by two independent observers.

### Measurement of TNF-α, IL-6, Malondialdehyde, and Lactate Dehydrogenase Levels

The levels of TNF-α and IL-6 in hippocampal tissues were detected by enzyme-linked immunosorbent assay (ELISA). The experiment was conducted according to the product instruction (ABclonal Technology Co., Ltd., Wuhan, China). The levels of MDA and LDH, which are the markers of lipid peroxidation, were determined using a thiobarbituric acid (TBA) assay kit and 2,4-dinitrophenylhydrazine colorimetric assay kit (Beyotime Technology, Shanghai, China) in accordance with the manufacturer’s protocol. After completing the drug treatments, hippocampal tissues of six mice in each group were collected and stored at −80°C until analysis. When performing the experiments, the tissues were homogenized in PBS buffer and centrifuged to collect the supernatant. The total protein content was tested using a Bicinchoninic Acid (BCA) protein assay kit (Solarbio, Beijing, China). The levels of TNF-α, IL-6, MDA and LDH was measured in nanomole per microgram (nmol/mg) of protein.

### Data Analysis

Data are presented as means ± standard error of mean (SEM). Analysis of variance (ANOVA) including one-way, two-way, and three-way ANOVA and mixed ANOVA (repeated-measures design) were used for the statistical analyses. Bonferroni’s *post hoc* test was performed to assess the differences between groups. Unpaired two-tailed Student’s *t*-test was used to compare two independent groups. The threshold for statistical significance was set at *P* < 0.05 (GraphPad, v.8.0, CA, United States).

## Results

### Animal Exclusion

A total of 390 C57BL/6 mice were initially purchased. With regard to accidental death after METH treatment, especially under HAT, the number of mice in each group was inconsistent in experimental design. In total, 24 mice died during METH treatments, 6 mice died because the failure of intragastric injection, and 5 mice were excluded due to poor general state and low activity in locomotion test. Thus, a total of 355 experimental animals were included in the data analysis.

### High Ambient Temperature Aggravated Methamphetamine-Induced Hyperthermia and Body Weight Loss

Mice were treated with four doses of 10 mg/kg METH with 2-h interval in between doses at normal (22°C) and high (28°C) ambient temperatures (Baladi et al., 2014), and the control mice received four saline (10 mg/kg) injections (Zhu et al., 2006; García-Cabrerizo et al., 2018). As shown in Figure 1B, METH exposure under HAT led to severe hyperthermia (left) and greater body weight loss (right). The mixed ANOVA (repeated measure) revealed significant effects on body temperature of METH treatment (*F*_1,54_ = 57.29, *P* < 0.001) and ambient temperature (*F*_1,54_ = 15.06, *P* < 0.001), and significant interaction of METH treatment with ambient temperature (*F*_1,54_ = 5.68, *P* = 0.021). In addition, the two-way ANOVA revealed significant main effects on body weight loss for METH treatment (*F*_1,54_ = 89.03, *P* < 0.001), ambient temperature (*F*_1,54_ = 19.86, *P* < 0.001), and interaction of METH treatment and ambient temperature (*F*_1,54_ = 17.01, *P* < 0.001). *Post hoc* comparisons indicated that the body weight loss was much more serious when METH was given under HAT (*P* < 0.001), compared to NAT.

### Methamphetamine Exposure Induced Depression-Like Behaviors in an Ambient Temperature and Dose-Dependent Manner

To investigate the potential effects of hyperthermia on depression-like behaviors, mice were treated with METH (10 mg/kg × 4 injections) at NAT (22°C) and HAT (28°C), and the behavioral tests including LMT, TST, and FST were performed 7 (T1) and 14 (T2) days later. As shown in Figure 1C, METH treatment under the NAT did not induce any depression-like behaviors in TST (T1: *P* = 0.782; T2: *P* = 0.390) and FST (T1: *P* = 0.442; T2: *P* = 0.365). However, METH treatment given with HAT induced depression-like behaviors both in TST (*P* < 0.001) and FST (*P* < 0.001) at 7 days after METH exposure (T1) but recovered in T2 tests (TST: *P* = 0.808; FST: *P* = 0.394). All the LMT results showed no significant differences between groups in T1 and T2 tests (NAT: *P* = 0.753 and *P* = 0.209; HAT: *P* = 0.456 and *P* = 0.853).

Next, we increased the doses of METH treatments to 10 mg/kg × 12 injections (in 3 days). The results of behavioral tests also showed no difference between groups in T1 (TST: *P* = 0.568; FST: *P* = 0.611) and T2 (TST: *P* = 0.707; FST: *P* = 0.938) tests when METH treatment was performed under NAT. However, METH treatment given with HAT induced long-lasting depression-like behaviors at least for 14 days. The results of FST and TST test revealed significant differences between saline and METH group in T1 (TST: *P* < 0.001; FST: *P* < 0.001) and T2 (TST: *P* < 0.001; FST: *P* < 0.001) tests. Moreover, METH treatments under NAT and HAT did not affect the locomotion of animals in T1 and T2 procedures (NAT: *P* = 0.749 and *P* = 0.979; HAT: *P* = 0.797 and *P* = 0.460).

Interestingly, we involved different METH exposure regimens and doses under NAT with the aim to induce depression-like behaviors. As shown in Figure 2B, long-term (a and b), higher dose (c), or escalating-dose (d) regimen of METH exposure failed to induce depression-like behaviors and locomotion deficit in mice, only except for the total distance traveled by mice in LMT, which increased after 7 or 14 days of METH treatments and 7 days of drug abstinence when compared to that of control mice (*P* < 0.001).

### Inhibition of Methamphetamine-Induced Hyperthermia and Depression-Like Behaviors Using Coral Calcium Hydride Generated Molecular Hydrogen

**FIGURE 3:**
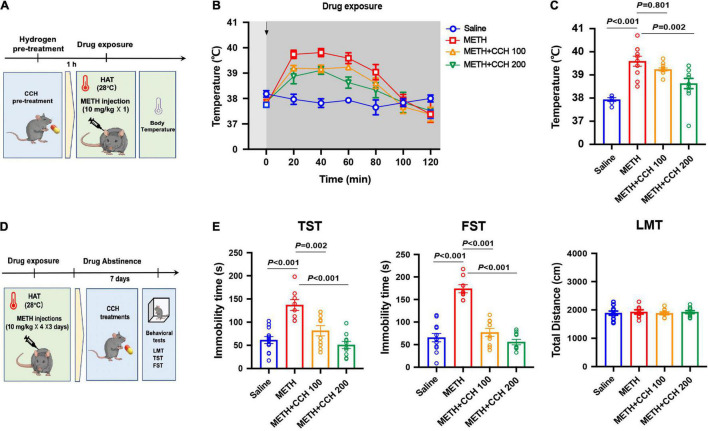
Coral calcium hydride (CCH) administration inhibited METH-induced hyperthermia and depression-like behaviors. **(A)** Timeline of CCH pre-treatment, METH exposure and core body temperature determination. **(B)** The core body temperature was determined every 20 min after METH (10 mg/kg, i.p.) injection for 2-h (*n* = 6, 10, 12, and 11). The arrows represent drug treatment. **(C)** The body temperature at 60 min after METH treatment was analyzed. **(D)** Timeline of METH exposure, therapeutic CCH treatment and behavioral tests. **(E)** Effect of CCH administration on METH-induced depression-like behaviors (*n* = 8 per group). Data are expressed as the mean ± SEM.

Since CCH interaction with water under acidic conditions leads to gradual HG production, mice were administrated with CCH intragastrically before and after METH treatments to investigate the preventative and therapeutic effect of molecular hydrogen on METH-induced hyperthermia and depression-like behaviors, respectively. Mice were pretreated with CCH (100 and 200 mg/kg, i.g.), and received METH injection (10 mg/kg, i.p.) 1-h later under HAT (28°C) (Figure 3A). The core body temperature was determined every 20 min for next 2-h. As shown in Figure 3B, CCH pretreatment significantly attenuated METH-induced hyperthermia. The two-way ANOVA indicated significant main effects on body temperature for drug treatment (*F*_3,35_ = 4.007, *P* = 0.015) and time (*F*_3.256,114_ = 35.58, *P* < 0.001), and interaction of drug treatment and time (*F*_18,210_ = 4.90, *P* < 0.001). Moreover, the one-way ANOVA revealed significant difference in body temperature at 60 min after METH treatment between groups (*F*_3,35_ = 14.06, *P* < 0.001). *Post hoc* comparisons indicated a rise in body temperature in METH-treated mice (*P* < 0.001), and an inhibitory effect of CCH pretreatment at doses of 200 mg/kg (*P* = 0.002), but not 100 mg/kg (*P* = 0.801) on METH-induced increase of body temperature (Figure 3C).

To examine the therapeutic effect of CCH on METH-induced depression, CCH (100 and 200 mg/kg, i.g.) treatments were given to mice for 7 days (once per day) after METH injections (10 mg/kg × 4 × 3 days, i.p.) (Figure 3D). As shown in Figure 3E, the results of one-way ANOVA revealed significant differences between groups in the immobility time in TST (*F*_3,28_ = 18.74, *P* < 0.001) and FST (*F*_3,28_ = 39.34, *P* < 0.001). *Post hoc* comparisons indicated significant therapeutic effect of 100 mg/kg (TST: *P* = 0.002; FST: *P* < 0.001) and 200 mg/kg (TST: *P* < 0.001; FST: *P* < 0.001) CCH on METH-induced depression-like behaviors. Furthermore, there were no differences noted on the total distance in LMT between groups (*F*_3,28_ = 1.473, *P* = 0.2432).

### Coral Calcium Hydride Induced Reversal of Methamphetamine-Induced Hippocampal Synaptic Plasticity Damage and Attenuation of Degree of Oxidative Stress and Neuroinflammation

**FIGURE 4:**
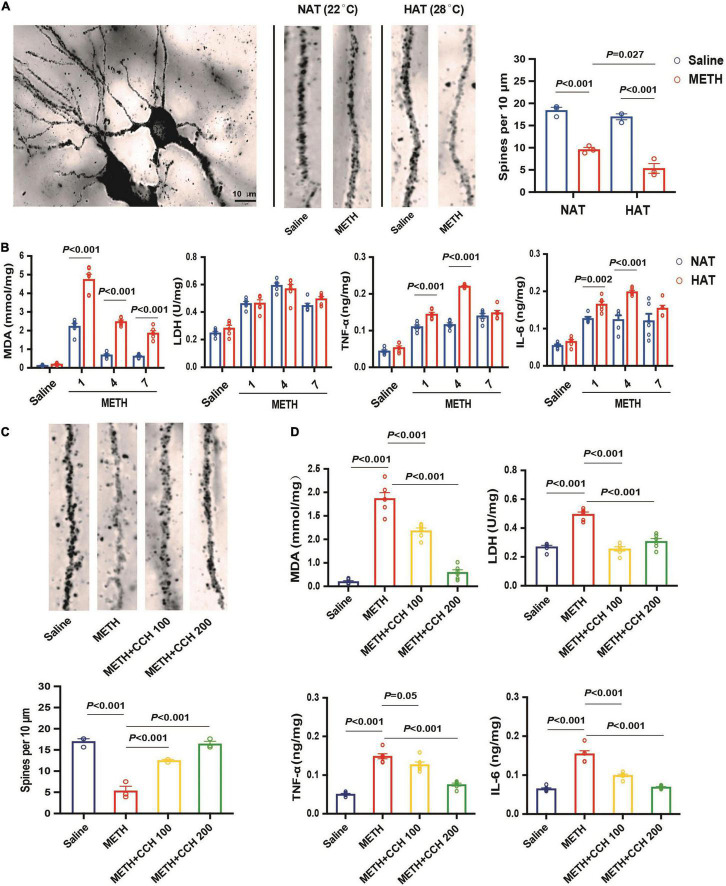
Coral calcium hydride administration inhibited METH-induced hippocampal synaptic plasticity damage, oxidative stress, and neuroinflammation. **(A)** HAT aggravated METH-induced hippocampal synaptic plasticity damage. The number of spines in hippocampal neuron was significantly decreased after METH treatment, especially when it given under HAT. Bar = 10 μm. **(B)** METH elicited time-dependent oxidative stress and neuroinflammation in hippocampus. HAT compounded METH-induced increase of MDA, TNF-α, and IL-6 levels, but did not change the activity of LDH. **(C)** CCH administration attenuated METH-induced hippocampal synaptic plasticity damage. **(D)** CCH administration inhibited METH-induced secretion of oxidative stress products and inflammatory cytokines in hippocampus. Data are expressed as the mean ± SEM (*n* = 3 per group in Golgi staining; *n* = 6 per group in measurement of MDA, LDH, TNF-α, and IL-6 levels).

Golgi staining showed that METH elicited hippocampal synaptic plasticity damage in mice, and this change was more severe when METH was given under HAT (*P* = 0.027, Figure 4A). The two-way ANOVA indicated significant main effects on spine number for METH treatment (*F*_1,8_ = 176.20, *P* < 0.001) and ambient temperature (*F*_1,8_ = 13.52, *P* = 0.006), but no interaction of METH treatment and ambient temperature (*F*_1,8_ = 3.353, *P* = 0.104). Furthermore, METH induced oxidative stress and neuroinflammation in a time-dependent manner. The one-way ANOVA indicated significant main effects on MDA levels (NAT: *F*_3,20_ = 271.0, *P* < 0.001; HAT: *F*_3,20_ = 137.8, *P* < 0.001), LDH (NAT: *F*_3,20_ = 90.15, *P* < 0.001; HAT: *F*_3,20_ = 26.56, *P* < 0.001), TNF-α (NAT: *F*_3,20_ = 69.17, *P* < 0.001; HAT: *F*_3,20_ = 196.2, *P* < 0.001), and IL-6 (NAT: *F*_3,20_ = 9,96, *P* < 0.001; HAT: *F*_3,20_ = 78.03, *P* < 0.001) for METH treatment. HAT aggravated METH-induced abnormal changes in MDA, TNF-α and IL-6 level (statistical data presented in Figure 4B). Interestingly, there was no difference between NAT and HAT group for the activity of LDH at each time point after METH treatments (Figure 4B). As shown in Figure 4C, the results of one-way ANOVA revealed significant differences between groups on the spine number (*F*_3,28_ = 18.74, *P* < 0.001). *Post hoc* comparisons indicated that CCH administration effectively reversed METH-induced hippocampal synaptic plasticity damage (CCH100: *P* < 0.001, CCH200: *P* < 0.001 compared to METH group). Similar results were revealed in MDA (*F*_3,20_ = 115.50, *P* < 0.001), LDH (*F*_3,20_ = 50.66, *P* < 0.001), TNF-α (*F*_3,20_ = 73.14, *P* < 0.001), and IL-6 (*F*_3,20_ = 81.24, *P* < 0.001) content measurement (Figure 4D). *Post hoc* comparisons also indicated an inhibitory effect of CCH administration on METH-induced increase of MDA (CCH100: *P* < 0.001, CCH200: *P* < 0.001), LDH (CCH100: *P* < 0.001, CCH200: *P* < 0.001), TNF-α (CCH100: *P* = 0.05, CCH200: *P* < 0.001), and IL-6 (CCH100: *P* < 0.001, CCH200: *P* < 0.001). Therefore, it can be stated conclusively that CCH administration significantly reversed hippocampal synaptic plasticity damage and alleviated oxidative stress and neuroinflammation induced by METH exposure.

## Discussion

Herein, we firstly demonstrated the role of hyperthermia in METH-induced depression-like behaviors in mice. The results of present study revealed that HAT (28°C) aggravates METH-induced hyperthermia and plays a key role in METH-induced depression-like behaviors. Secondly, we further clarified the effect of CCH, on METH-induced hyperthermia and behavioral abnormality. Treatment with CCH significantly inhibited METH-induced hippocampal synaptic plasticity damage and attenuated the rise in oxidative stress products and inflammatory cytokines in the hippocampus. Therefore, our study suggested that CCH used as an efficient hydrogen-rich agent is a novel and effective treatment of METH-induced psychiatric disorders.

It is known that frequent use of psychostimulants causes elevated behavioral and cognitive activity, and can also lead to severe psychiatric symptoms (King et al., 2010). The symptoms, such as psychosis, depression, and anxiety, are predictive of drug relapse, and significantly increase the risk of suicidal behavior and mortality (Archer et al., 2018). Previous studies have revealed that withdrawal of amphetamine-type psychostimulants and major depressive disorder share remarkable behavioral similarities in humans (Durdle et al., 2008; Härtel-Petri et al., 2017). Also, withdrawal from chronic or sub-chronic METH exposure induces anxiety and depression-like behaviors in several animal models (McCoy et al., 2011; North et al., 2013). The dose and duration of chronic METH usage range from 2 to 10 mg/kg and 7 days to 8 weeks, respectively (Ding et al., 2020). In addition, a binge dose (5–10 mg/kg × 4 at 2 h intervals) and the acute bolus drug administration (20–40 mg/kg) of METH have been employed frequently to study its neurotoxicity (Zhu et al., 2006; Melega et al., 2007). Fonseca et al. (2017) reported that a single neurotoxic dose of METH (30 mg/kg) induced a long-lasting depressive behavior in mice. However, the present study found that multiple repeated 10 mg/kg METH-injections paradigm induced depression-like behaviors in an ambient temperature and dose-dependent manner. We also considered that METH-induced hyperthermia is an important factor for the occurrence of depression-like behaviors. In a previous study, the author defined “neutral” as 24–27°C and “warm” as 28–37°C (Sabol et al., 2013). Indeed, 28°C cannot be considered as a high ambient temperature, but we found that most animals died after METH exposure when the ambient temperature was higher than 28°C. Therefore, we defined 28°C as the high ambient temperature in the present study, and the results revealed that HAT (28°C) aggravated METH-induced hyperthermia and lead to depression-like behaviors. No significant alteration in total distance in open field test indicates that METH induced depression-like behaviors do not affect the locomotor activity. Interestingly, the same METH-injections paradigm under NAT (22°C) and chronic METH treatments (10 mg/kg, 7 or 14 days) or escalating-dose (2 ∼ 15 mg/kg, 3 days) of METH exposure failed to induce depression-like behaviors. However, the difference in timescale of behavior tests after METH withdrawal also was a key factor related to the contradictory behavioral results. Indeed, a major pitfall of the present study is that it did not observe the earlier depression-like behaviors (less than 7 days) in these METH-treated mice.

Molecular hydrogen, as a selective antioxidant, was first reported by Ohsawa et al. (2007). Up to now, it has been shown to exhibit distinct potential as a novel therapeutic agent for a wide range of diseases, especially oxidative stress-mediated diseases. We previously found that molecular hydrogen significantly attenuated anxiety-like behaviors in morphine-withdrawn mice, and it was able to inhibit the acquisition and transfer of low dose METH-induced behavioral sensitization to a certain extent (Wen et al., 2020). Drinking or bathing with hydrogen-rich water and inhalation of HG were popular methods used to administer hydrogen to humans, while hydrogen-rich saline injection, *ad libitum* hydrogen-rich water consumption, and inhalation of HG were common methods to deliver molecular hydrogen to animals in experimental research. However, due to the low solubility of hydrogen in water, it is not easy to realize high concentration and long-term accumulation by hydrogen-rich water consumption or hydrogen-rich saline injection to animal model in a particular point of time. Therefore, we only evaluated the inhibitory effect of hydrogen on low dose METH-induced behavioral abnormality and neurotoxicity. Recent studies demonstrated that the hydrogen level released by CCH administration was more reliable and robust than hydrogen-rich water *in vitro* and *in vivo*. Moreover, Ueda et al. (2011) reported that CCH exerted antioxidant activity by enhancing the basal endogenous antioxidant ability in the hippocampus of rats. Consistent with above results, we found that CCH administration significantly attenuated large dose repeated METH treatment-induced severe hyperthermia and inhibited depression-like behaviors in FST and TST without altering the locomotion in mice. There has a previous study reported that molecular hydrogen potentiates hypothermia and prevents hypotension and fever in LPS-induced systemic inflammation. They found that molecular hydrogen caused a reduction in surges of TNF-α, IL-1β, and prostaglandin E2 (PGE2) in plasma and exerted anti-inflammatory effects strong enough to prevent fever by altering hypothalamic PGE2 production (Saramago et al., 2019). As already known, depressed mood and anhedonia are core symptoms of major depressive disorder (Rizvi et al., 2016). FST and TST are common tests used for assessing despair-like behaviors in laboratory animals (Zhao et al., 2019). In addition to above results, we investigated the effect of METH exposure and CCH treatment on anhedonia-like behavior, and consistent results were revealed in sucrose preference test (see Supplementary Figure 1). It is worthy to note that most studies in the past used oral intake of CCH or CCH-rich diet feeding for CCH administration. However, due to the restriction of time for drug treatment and body temperature determination, intragastric CCH treatment was involved in the present study. Meanwhile, we considered that long-term administration of intragastric injection might be a stress to the animals and would create influence on behavioral test, so intragastric CCH treatment was only given for 7 days and then the behavioral tests were subsequently performed. We also determined the effects of CCH treatment on locomotion and depression-like behaviors, as well as the index of oxidative stress and inflammation in hippocampus of naïve mice (see Supplementary Figure 2). Furthermore, coral calcium (CC) was used as a controlled treatment to exclude the therapeutic effects of other ingredients (see Supplementary Figure 3), and the result showed that administration of CC did not affect METH-induced depression-like behaviors. The previous data also revealed that there were no changes in the serum total Ca^2+^ levels after CCH treatment (Hou et al., 2016). Therefore, we considered that the protective effect of CCH was dependent on molecular hydrogen derived from CCH instead of other ingredients.

Although depressive symptoms and METH withdrawal have common neurobiological symptoms, our search for possible mechanisms of METH-induced depression-like behavior was focused on the METH-induced neurotoxicity and the involvement of hippocampal synaptic plasticity damage (Ren et al., 2017; Papageorgiou et al., 2019; Golsorkhdan et al., 2020). Recent studies have found that hippocampal volume and neuron loss are prominent characters of depression (Barch et al., 2019; Sheline et al., 2019). Accumulating evidence also supports the existence of alteration of hippocampal synaptic plasticity in depressive symptoms (Lu et al., 2014; Liu et al., 2018). Therefore, it has been proposed that increase in METH-induced oxidative stress products and inflammatory factors, results in decreased synaptic proteins synthesis and structural damage, ultimately leading to depressive symptoms (Huang et al., 2013). Likewise, HAT aggravated hyperthermia, and it also induced remarkable increase of MDA, LDH, IL-6, and TNF-α in hippocampus of METH-treated mice. Golgi staining showed corresponding severe damage in hippocampal synaptic plasticity when METH was given under HAT. In addition, CCH administration significantly reduced the damage of METH-induced hippocampal synaptic plasticity and reduced the levels of MDA, LDH, IL-6, and TNF-α in hippocampus of mice.

However, there have two limitations of the present study. Firstly, although the present data revealed the inhibitory effect of CCH on METH-induced severe hyperthermia, whether it affect the hypothalamus or generate peripheral effect thereby causing temperature change in METH-treated mice should be explored. Moreover, since the evidence of CCH toxicity in humans is limited, the possible toxicity should be carefully investigated in future studies.

## Conclusion

In the present study, we revealed that hyperthermia plays a key role in METH-induced depression-like behaviors. In addition, it was also proved that molecular hydrogen released by CCH effectively ameliorated METH-induced hyperthermia and depression-like behaviors, and this function was possibly elaborated *via* the regulation of hippocampal synaptic plasticity damage mediated by oxidative stress and neuroinflammation. In summary, based on our present study, it can be concluded that CCH acts as a protective antioxidant treatment and may have potential application in reducing the risk of psychiatric symptoms in METH abusers.

## Data Availability Statement

The original contributions presented in the study are included in the article/Supplementary Material, further inquiries can be directed to the corresponding authors.

## Ethics Statement

The animal study was reviewed and approved by Hebei Medical University.

## Author Contributions

DW and CM designed the experimental plans. XW, BT, and RH performed the behavioral part of the study. CH and ZZ did the molecular biological experiment in text. LZ and BX analyzed data. ZN and BC wrote and revised the manuscript. All authors contributed to the article and approved the submitted version.

## Conflict of Interest

The authors declare that the research was conducted in the absence of any commercial or financial relationships that could be construed as a potential conflict of interest.

## Publisher’s Note

All claims expressed in this article are solely those of the authors and do not necessarily represent those of their affiliated organizations, or those of the publisher, the editors and the reviewers. Any product that may be evaluated in this article, or claim that may be made by its manufacturer, is not guaranteed or endorsed by the publisher.

## Supplementary Material

Supplementary Figure 1 Administration of coral calcium hydride (CCH) inhibited methamphetamine (METH)-induced depression-like behavior in sucrose preference test. METH treatment and CCH administration were the same as the procedure presented in Figure 3D. After 7 days of CCH administration, sucrose preference test was conducted to assess the anhedonia of mice. Two bottles of 1% (w/v) sucrose solution were provided in each cage to conduct 48 h habituation. Then, mice were deprived of water for 24 h and individually housed before being exposed to sucrose bottle and water bottle for 24 h, and sucrose and water intake were measured. Sucrose preference was calculated as the percentage of sucrose consumption in total liquid consumption. METH exposure under HAT induced the decrease of sucrose intake, and CCH administration significantly inhibited the effect of METH (*n* = 8 per group). Data are expressed as the mean ± SEM.

Supplementary Figure 2 Administration of CCH without METH treatment did not induce the depression-like behavior and the activation of oxidative stress and neuroinflammation in hippocampus. **(A)** Timeline of CCH treatment, behavioral test and tissue collection. **(B)** Administration of CCH without METH did not induce the depression-like behavior (*n* = 8 per group). **(C)** Administration of CCH without METH treatment has no effect on the activity of LDH and the content of MDA, TNF-α, and IL-6 in hippocampus (*n* = 6 per group). Data are expressed as the mean ± SEM.

Supplementary Figure 3 Administration of coral calcium (CC) did not affect METH-induced depression-like behaviors. **(A)** Timeline of CC/CCH treatment and behavioral tests. **(B)** Administration of CC after METH treatment did not alter the depression-like behavior. Mice were administered with CC or CCH (200 mg/kg, intragastric route [i.g.]) resuspended in 0.2 mL pure water twice per day after METH exposure (*n* = 6, 6, 8, and 6). Data are expressed as the mean ± SEM.
