# Supplementary figures and images for: The Role of Hyperthermia in Methamphetamine-Induced Depression-Like Behaviors: Protective Effects of Coral Calcium Hydride
Source: Front Mol Neurosci. 2022 Jan 4;14:808807. doi: 10.3389/fnmol.2021.808807 (PMC8764150; doi:10.3389/fnmol.2021.808807)

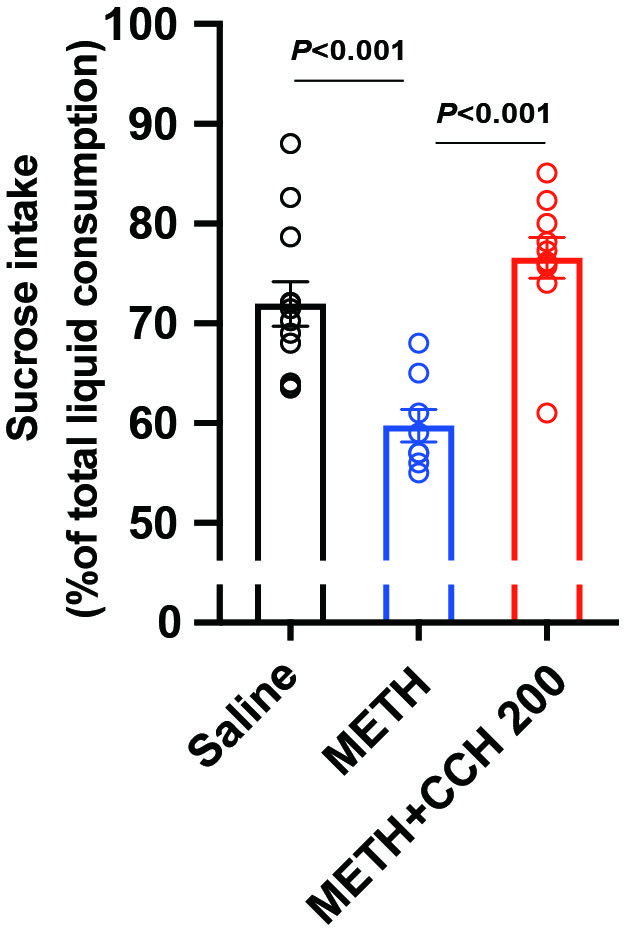

Supplement: Supplementary Figure 1 — Administration of coral calcium hydride (CCH) inhibited methamphetamine (METH)-induced depression-like behavior in sucrose preference test. METH treatment and CCH administration were the same as the procedure presented in Figure 3D. After 7 days of CCH administration, sucrose preference test was conducted to assess the anhedonia of mice. Two bottles of 1% (w/v) sucrose solution were provided in each cage to conduct 48 h habituation. Then, mice were deprived of water for 24 h and individually housed before being exposed to sucrose bottle and water bottle for 24 h, and sucrose and water intake were measured. Sucrose preference was calculated as the percentage of sucrose consumption in total liquid consumption. METH exposure under HAT induced the decrease of sucrose intake, and CCH administration significantly inhibited the effect of METH (n = 8 per group). Data are expressed as the mean ± SEM. [file Image_1.JPEG]

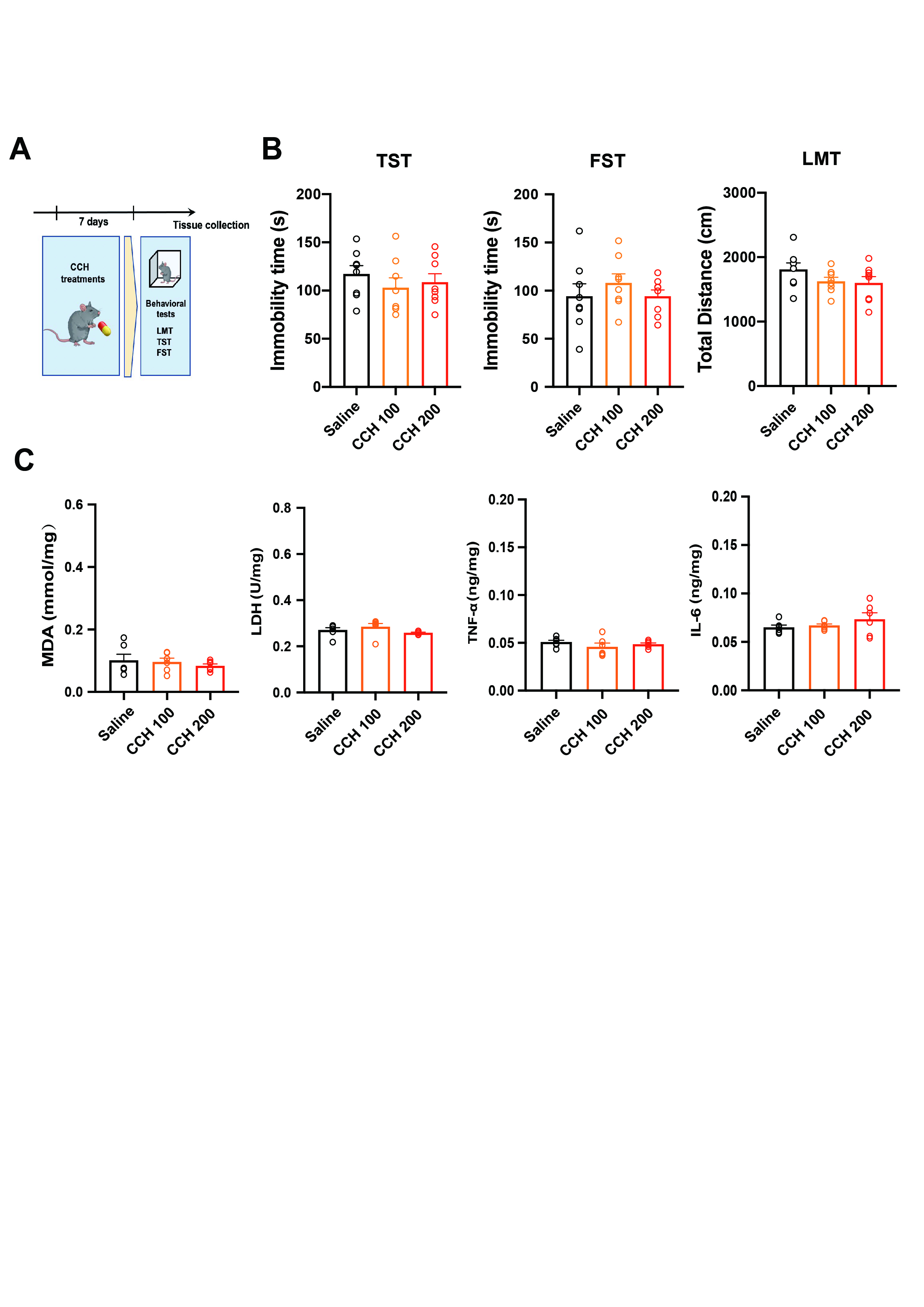

Supplement: Supplementary Figure 2 — Administration of CCH without METH treatment did not induce the depression-like behavior and the activation of oxidative stress and neuroinflammation in hippocampus. (A) Timeline of CCH treatment, behavioral test and tissue collection. (B) Administration of CCH without METH did not induce the depression-like behavior (n = 8 per group). (C) Administration of CCH without METH treatment has no effect on the activity of LDH and the content of MDA, TNF-α, and IL-6 in hippocampus (n = 6 per group). Data are expressed as the mean ± SEM. [file Image_2.jpg]

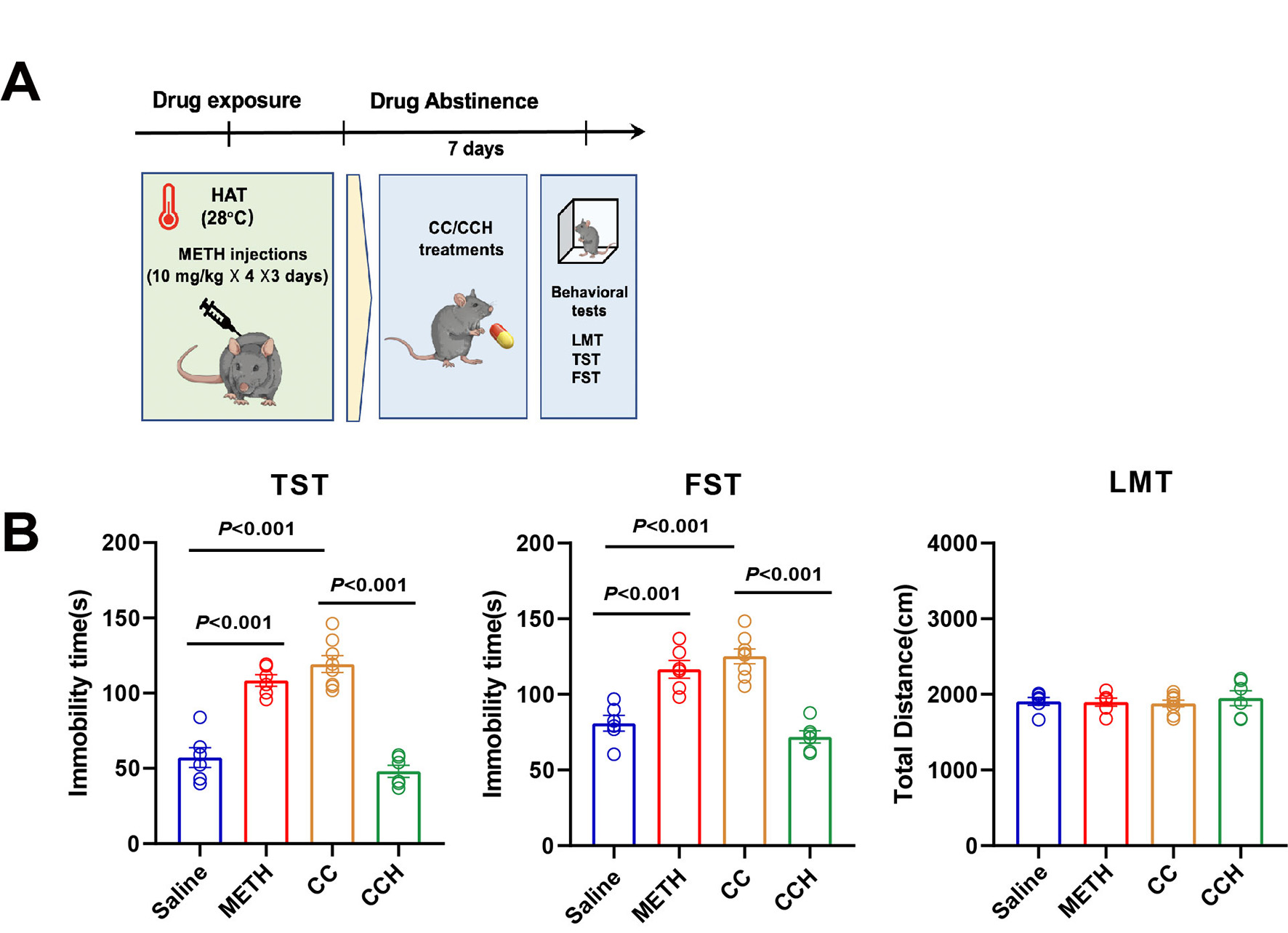

Supplement: Supplementary Figure 3 — Administration of coral calcium (CC) did not affect METH-induced depression-like behaviors. (A) Timeline of CC/CCH treatment and behavioral tests. (B) Administration of CC after METH treatment did not alter the depression-like behavior. Mice were administered with CC or CCH (200 mg/kg, intragastric route [i.g.]) resuspended in 0.2 mL pure water twice per day after METH exposure (n = 6, 6, 8, and 6). Data are expressed as the mean ± SEM. [file Image_3.JPEG]
